# The acidic domains of the Toc159 chloroplast preprotein receptor family are intrinsically disordered protein domains

**DOI:** 10.1186/1471-2091-10-35

**Published:** 2009-12-30

**Authors:** Lynn GL Richardson, Masoud Jelokhani-Niaraki, Matthew D Smith

**Affiliations:** 1 Department of Biology, Wilfrid Laurier University, Waterloo, ON, N2L 3C5, Canada; 2 Department of Biology, University of Waterloo, Waterloo, ON, N2L 3G1, Canada; 3 Department of Chemistry, Wilfrid Laurier University, Waterloo, ON, N2L 3C5, Canada; 4 Current address: Department of Molecular and Cellular Biology, University of Guelph, Guelph, ON, Canada

## Abstract

**Background:**

The Toc159 family of proteins serve as receptors for chloroplast-destined preproteins. They directly bind to transit peptides, and exhibit preprotein substrate selectivity conferred by an unknown mechanism. The Toc159 receptors each include three domains: C-terminal membrane, central GTPase, and N-terminal acidic (A-) domains. Although the function(s) of the A-domain remains largely unknown, the amino acid sequences are most variable within these domains, suggesting they may contribute to the functional specificity of the receptors.

**Results:**

The physicochemical properties of the A-domains are characteristic of intrinsically disordered proteins (IDPs). Using CD spectroscopy we show that the A-domains of two *Arabidopsis *Toc159 family members (atToc132 and atToc159) are disordered at physiological pH and temperature and undergo conformational changes at temperature and pH extremes that are characteristic of IDPs.

**Conclusions:**

Identification of the A-domains as IDPs will be important for determining their precise function(s), and suggests a role in protein-protein interactions, which may explain how these proteins serve as receptors for such a wide variety of preprotein substrates.

## Background

Most chloroplast proteins are encoded in the nucleus and translated in the cytosol with an N-terminal transit peptide that facilitates recognition by receptors of the Toc complex. In *Arabidopsis*, two families of GTPases are responsible for preprotein recognition; the Toc34 and Toc159 receptors [1-6]. Toc159 interacts with transit peptides [6] at early stages of import [2,7], suggesting that it is the primary preprotein receptor. However, it is unknown precisely how this receptor recognizes preproteins, and its function in subsequent preprotein translocation remains unclear. There are four Toc159-related proteins in *Arabidopsis*: atToc159, -132, -120 and -90 [8,9]. These receptors are able to distinguish between semi-distinct classes of substrates; atToc159 is implicated in the import of photosynthetic proteins, while atToc132 and atToc120 appear to be functionally redundant, and are primarily involved in the import of non-photosynthetic proteins [6,8,10-12]. The Toc159 receptors have three distinguishable regions: an N-terminal acidic (A-) domain and a central GTPase (G-) domain, which extend into the cytosol, and a C-terminal membrane (M-) domain that anchors the protein to the outer chloroplast membrane [8,13]. The G- and M-domains of the *Arabidopsis *family members share ~65% sequence identity [8,10]. The G-domain is involved in targeting Toc159 to the chloroplast during initial Toc complex assembly [14-16], comprises at least part of a transit peptide binding site [6], and acts as part of a GTP-regulated switch for preprotein recognition [7,17]. Less information is available regarding the A- and M-domains. The A-domain is highly variable in amino acid sequence between species and among the Toc159 family members in *Arabidopsis *(~20% identity) and has no known conserved functional motifs [8,10]. Although it appears to be non-essential for Toc159 function [13,17,18], the A-domain has been hypothesized to confer differential substrate recognition, owing to the variability in amino acid sequence among family members [8], and evidence has recently been presented that the Toc159 A-domain interacts with actin [19]. Despite reports on its dispensability for Toc159 function, the size of the A-domain (it accounts for almost 50% of the length of Toc159) suggests that it is likely to confer some important function(s) to the receptor.

Based on hydrophobic cluster analysis of its A-domain, Toc159 has been proposed to belong to a growing class of natively unstructured or intrinsically disordered proteins (IDPs) [20], which show lack of globular structure over their entire length or contain large unstructured regions [21], and have been estimated to account for up to ~30% of all proteins in higher eukaryotes [21,22]. Several notable characteristics of the Toc159 family A-domains are consistent with their classification as IDPs. They possess a high number of charged (acidic) amino acid residues, have a repetitive amino acid sequence, demonstrate aberrant mobility during SDS-PAGE and are highly sensitive to proteolysis [4,13,20,23-26]. In addition, IDPs are known to undergo extensive post-translational modification, and in particular, are enriched in phosphorylation sites [21]. Consistent with this observation, the A-domain of Toc159 was recently identified in a proteomic survey of phosphorylated *Arabidopsis *proteins [27].

IDP domains are involved in highly dynamic protein-protein interactions [21,22], often of high specificity and low affinity, and may interact with many different binding partners, including IDP regions of other proteins. During such interactions, IDPs often undergo induced folding, which has been proposed to explain how they are able to achieve specific, yet low affinity interactions with multiple binding partners [21,28]. In the current study, CD spectroscopy was used to demonstrate that the A-domain of two members of the *Arabidopsis *Toc159 family, atToc159 and atToc132, are IDPs. This represents the first investigation into the structure of the A-domains of the Toc159 family, and has implications for future studies aimed at understanding the function of these domains, and the role of Toc159 receptors in general, in chloroplast protein import.

## Results

### A-domains are predicted to be natively unfolded

**Figure 1:**
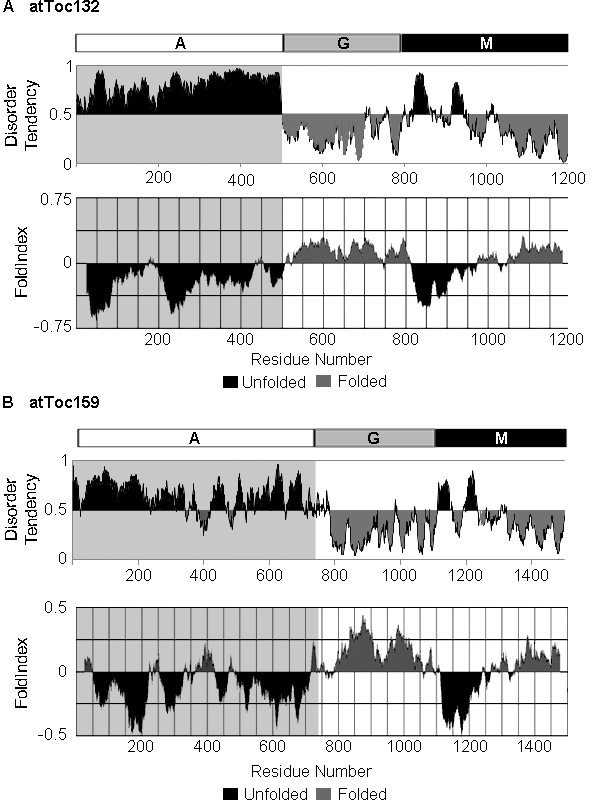
**The A-domains of atToc159 and atToc132 are predicted to be largely disordered**. IUPred [30] (top panels) and FoldIndex [29] (lower panels) were used for disorder predictions of full-length atToc132 (A) and atToc159 (B). The amino acid numbers of the A-, G- and M- domain boundaries are indicated. The regions predicted to be disordered are shaded in dark grey.

A recent study led to the suggestion that the A-domain of atToc159 may be natively unfolded [20]. In the current study, disorder within atToc132 (AGI# At2g16640) and atToc159 (AGI# At4g02510) was predicted using FoldIndex [29] and IUPred [30]. Delineation of the A-domains was designated as previously described [10]. Both programs predict the A-domains of atToc132 and atToc159 (residues 1-455 and 1-727, respectively) to be mainly unfolded (Figure 1). The A-domains of atToc120 (AGI# At3g16620) and Toc159 from *Pisum sativum *(psToc159) (Accession AAF75761) are also predicted to be largely disordered (data not shown). A-domains of atToc159 and atToc132 were selected as representatives for further examination.

### Expression and purification of 132A_His _and 159A_His_

**Figure 2:**
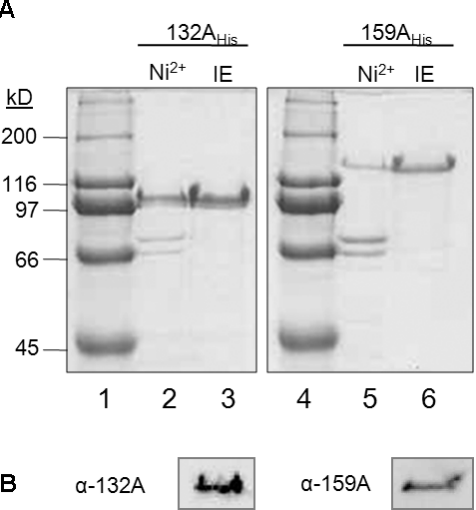
**Expression and purification of recombinant A-domains of atToc159 and atToc132**. (A) N-terminally His_6_-tagged versions of atToc132 (132A_His_) and atToc159 (159A_His_) were expressed in *E. coli*, purified using Ni^2+^-NTA chromatography (Ni^2+^, lanes 2 & 5) and ion exchange (IE, lanes 3 & 6), and analyzed using SDS-PAGE stained with Coomassie blue. Molecular weight markers (kD) are indicated (lanes 1 & 4). (B) Identity of the purified proteins was confirmed by Western blotting with antibodies against the A-domains of atToc132 (α-132A) and atToc159 (α-159A).

*E. coli*-expressed A-domains of atToc132 and atToc159 possessing N-terminal His_6 _tags (132A_His _and 159A_His_) were purified using Ni^2+^-charged resin (Figure 2A, lanes 2 and 5). To gain a level of purity suitable for CD spectroscopy, the proteins were further purified by ion exchange (Figure 2A, lanes 3 and 6), and the identities of the ion-exchange purified proteins were confirmed by Western blotting (Figure 2B). The theoretical molecular weights of 132A_His _and 159A_His _are ~50 kDa and ~76 kDa, respectively; however, these proteins migrate at an apparent molecular weight approximately 50 kDa larger than expected during SDS-PAGE (Figure 2A). The same phenomenon has been observed for full-length Toc159 [13,18]; however, when the A-domain is proteolytically degraded, the remainder of the protein (G+M domains) migrates as expected [13,18]. Aberrant electrophoretic mobility is characteristic of acidic proteins [31], and is a common property of IDPs [26].

### Structural analysis of 132A_His _and 159A_His _using CD spectroscopy

**Figure 3:**
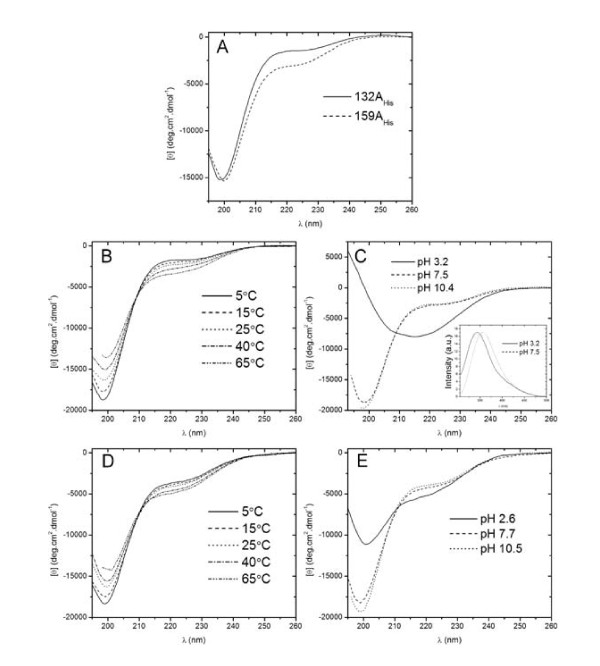
**Purified recombinant A-domains of atToc159 and atToc132 were analysed using circular dichroism (CD) and fluorescence spectroscopy**. (A) Far-UV CD spectra of 132A_His _and 159A_His _at 25°C and pH 8.0. Temperature-dependent and pH-dependent far-UV CD spectra of 132A_His _(B and C) and 159A_His _(D and E) are also shown. Summary of the deconvoluted data is shown in Table 1. Intrinsic fluorescence of 132A_His _excited at 295 nm was measured at pH 3.2 and 7.5 (inset, panel C).

CD spectroscopy was used to assess the secondary structure content of 132A_His _and 159A_His_. Under non-denaturing conditions both proteins show far-UV spectra typical of unfolded proteins, characterized by the presence of a deep minimum in the vicinity of 200 nm and a relatively low ellipticity at ~220 nm (Figure 3A) [32]. Spectra were deconvoluted, revealing the presence of 76% and 63% random coil secondary structure in 132A_His _and 159A_His_, respectively (Table 1). This indicates that at physiological temperature and pH, 132A_His _and 159A_His _are mainly disordered, supporting the hypothesis that the A-domains are IDPs.

**Table 1:** Secondary structure composition of 159A_His _and 132A_His _under different experimental conditions^a^

Protein and experimental condition	α-helix	β-sheet	Random coil
132A 25°C, pH 8	0.03	0.22	0.76
132A 5°C (pH 8)	0.04	0.33	0.63
132A 60°C (pH 8)	0.07	0.43	0.49
132A pH 3 (25°C)	0.25	0.20	0.55
132A pH 10 (25°C)	0.03	0.21	0.76
132A 10% TFE	0.04	0.30	0.66
132A 50% TFE	0.28	0.28	0.44
			
159A 25°C, pH 8	0.05	0.32	0.63
159A 5°C (pH 8)	0.04	0.31	0.65
159A 60°C (pH 8)	0.07	0.38	0.55
159A pH 3 (25°C)	0.08	0.41	0.51
159A pH 10 (25°C)	0.04	0.22	0.75
159A 10% TFE	0.08	0.40	0.52
159A 50% TFE	0.28	0.20	0.52

^a^Deconvolution of the far-UV CD spectra for 159A_His _and 132A_His _was performed using the K2D method. Values represent the proportion of secondary structure composition.

### Effects of temperature and pH on A-domain structure

To further characterize the structural properties of the A-domains, the effects of temperature and pH on the conformation of 132A_His _and 159A_His _were investigated. Both 132A_His _and 159A_His _exhibit a modest temperature-induced gain in secondary structure, as shown by an increase in negative ellipticity at ~220 nm with increasing temperature (Figure 3B, D). Spectra deconvolution reveals that the α-helical content of 159A_His _increases from 5% to 7%, and β-sheet content increases from 32% to 38% at 65°C as compared to 25°C, coinciding with a decrease in random coil content from 63% to 55% (Table 1). Random coil content of 132A_His _also decreases from 76% (at 25°C) to 49% (at 65°C), and again, there is a concomitant gain in β-sheet content from 22% to 43%, and in α-helical content from 3% to 7% (Table 1). Such gains in secondary structure with increasing temperature are characteristic of IDPs, and are in contrast to the typical loss of structure associated with the heating of globular proteins [26,32]. In addition, both proteins gain considerable overall secondary structure at low pH (Figure 3C, E). Specifically, 159A_His _contains 51% random coil, 8% α-helix and 41% β-sheet at pH ~3, compared to 63% random coil, 5% α-helix and 32% β-sheet at neutral pH. Likewise, 132A_His _contains 55% random coil, 25% α-helix and 20% β-sheet at pH ~3 compared to 76% random coil, 3% α-helix and 22% β-sheet at neutral pH (Table 1). These increases in secondary structure at low pH may be attributed to a decrease in net charge at a pH below their respective theoretical pI values of 4.25 (132A_His_) and 4.0 (159A_His_). Presumably, a decrease in net charge leads to a decrease in electrostatic repulsion between negatively charged residues, allowing for partial folding. An increase in structure for 132A_His _at low pH can also be detected using fluorescence spectroscopy (Figure 3C, inset). 132A_His _contains two Trp residues (residues 225 and 234) that fluoresce when excited at 295 nm (159A_His _does not contain Trp, so was not analyzed using fluorescence spectroscopy). The fluorescence maximum of 132A_His _shifts to a lower wavelength at pH 3, suggesting that the Trp residues are less exposed to solvent as a result of partial folding at low pH - a commonly observed phenomenon of acidic IDPs [26,32].

### Trifluoroethanol induces structure of A-domains

**Figure 4:**
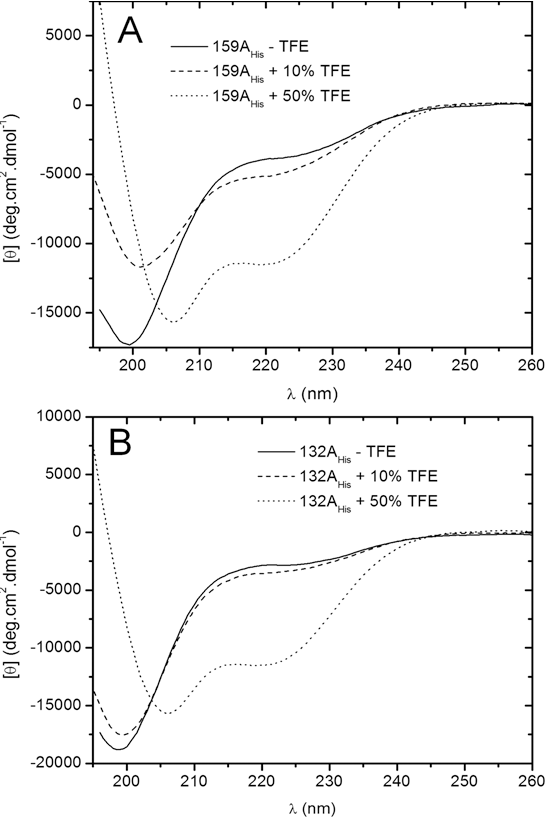
**132A_His _and 159A_His _gain structure in the presence of trifluoroethanol (TFE)**. Far-UV CD spectra of 159A_His _(A) and 132A_His _(B) in the absence (-TFE) and presence of 10% and 50% TFE at 25°C. A summary of the deconvoluted data is shown in Table 1.

In the presence of trifluoroethanol (TFE) both 132A_His _and 159A_His _show a notable increase in secondary structure from ~3-5% α-helix, 22-32% β-sheet in the absence of TFE to 28% α-helix, 28% β-sheet in 50% TFE (Figure 4, Table 1). These results, as well as the behaviour of the proteins at temperature and pH extremes, highlight the conformational flexibility of the A-domains and indicate they have the ability to form secondary structure depending on their environment. This conformational flexibility may reflect an ability to undergo conformational changes as part of their physiological function, for example during ligand binding. Several IDPs are noted for their ability to undergo significant conformational changes upon binding to their substrates (reviewed in [21]).

Interestingly, the A-domains showed differences in the amount and types of secondary structure gained at low pH and in the presence of 50% TFE. In particular, 132A_His _gained more structure than 159A_His _under these conditions. These differences in conformational flexibility could reflect functional differences between the A-domains of atToc132 and atToc159.

## Discussion

As part of an evolutionary study into the origin of Toc159, it was suggested that the A-domain of atToc159 might be natively unfolded [20]. In the current study, we decided to take a structural approach to investigate this possibility in more detail, to gain insight into the function of the A-domain. We started by using disorder prediction programs that strongly predicted the A-domains of atToc132 and atToc159 (as well as atToc120 and psToc159) to be unstructured. In agreement with these predictions, the A-domains were shown experimentally to be disordered under non-denaturing conditions, and underwent structural changes characteristic of IDPs at extremes of temperature and pH. Furthermore, in the presence of 50% TFE, both A-domains gained considerable structure, which together with the effects of extreme temperature and pH, shows that the proteins have the propensity to shift to a more ordered state under certain conditions, which could include association with binding partners. Overall, the data presented here are consistent with the classification of the A-domains as intrinsically disordered protein domains. To date, the function of the A-domains remains largely unknown, with the exception of the recently suggested role in binding to actin filaments [19], thus the identification of the Toc159 family A-domains as IDPs has several potential implications for its function. In general, IDPs have a large surface area under physiological conditions allowing them to interact with several binding partners simultaneously [26]. Indeed, the A-domain accounts for almost 50% of the total length of atToc159, which represents a large surface area available for multiple protein-protein interactions. While binding partners of the A-domain other than actin have not yet been identified, several candidates exist. For example, the A-domain may interact with other components of the Toc complex (i.e. Toc33/34 and/or Toc75) to help facilitate complex assembly, a function previously reported for IDPs [33]. This function is reminiscent of the proposed role for the N-terminal unstructured domain of the yeast peroxisomal import receptor Pex5p in stabilization of the import complex [34]. Alternatively, the A-domain may not possess intrinsic bioactivity, but be involved in the regulation of the GTPase activity of the adjacent Toc159 G-domain, or that of the other Toc GTPase, Toc33/34. Modulation and/or regulation of adjacent, globular functional domains has been previously observed for N-terminally located IDP regions [35].

Finally, perhaps the most intriguing potential function for the A-domain that emerges from the finding that it is an IDP is a role in transit peptide recognition. Transit peptides are variable in length (typically 50-70 amino acids) and sequence, are rich in hydroxylated amino acids, scarce in acidic amino acids, and lack a defined three-dimensional structure in aqueous solution [36-39]. It is unknown precisely how they are recognized by receptors of the Toc complex; however, it has been shown that subgroups of transit peptides contain distinct motifs that affect their import efficiency and receptor specificity [12,40-43]. Therefore, it is interesting to speculate that the disordered nature of the A-domains may facilitate interactions with multiple motifs within transit peptides, allowing for differential recognition of preproteins. The differences in structural dynamics observed between 159A_His _and 132A_His _in this study may be reflective of such an ability to discriminate between preproteins. In addition, it has been proposed that IDPs with large surface area may act in a "fly-casting" mechanism to increase the speed of low affinity protein-protein interactions [44], which would allow for preproteins to be efficiently passed to downstream components of the chloroplast protein import apparatus. Such transient, low affinity interactions would be consistent with the reversible, energy-independent binding of preproteins to chloroplasts at the initial stages of import [2,5], and may also partially explain the inability to detect A-domain-preprotein interactions *in vitro *[6], as well as discrepancies observed in the order of preprotein binding to Toc34 and Toc159 [1,45]. Analogously, the Tom70 receptor of the yeast mitochondrial protein import apparatus contains a disordered region that possesses multiple interaction sites for its mitochondrial protein substrates [46,47].

While interactions between the Toc159 family A-domains and transit peptides is an attractive mechanism for transit peptide recognition, detection of transient protein-protein interactions is technically challenging, and it is not yet clear whether the techniques employed here will be sufficient to detect potential interactions between the A-domain and transit peptides. CD may prove useful in this regard if association is accompanied by a disorder-to-order transition [26]; the acquisition of α-helical structure in the presence of TFE suggests that the A-domain has a propensity to do so (Figure 4). Techniques such as surface plasmon resonance, isothermal titration calorimetry, and nuclear magnetic resonance may also prove useful in future attempts to test whether such (transient) interactions take place; some of these techniques have been used in studies on one of the best characterized IDPs, the phosphorylated kinase inducible activation domain (pKID), and others [26,48,49].

## Conclusions

In summary, the A-domain represents a large portion of the Toc159 receptors and differs significantly among members of this family. The function(s) of this domain, however, has remained elusive. In this study, the structure of the A-domains has been investigated for the first time. The finding that the A-domains are intrinsically disordered has implications for understanding their function(s), and future studies on the Toc159 receptors will be aimed at identifying A-domain binding partners to help elucidate the role of this domain in chloroplast protein import.

## Methods

### Cloning, expression, and purification of 132A_His _and 159A_His_

The first 1365 or 2181 basepairs of the *atTOC132 *and *atTOC159 *cDNAs, which correspond to the A-domains of atToc132 (132A) and atToc159 (159A), respectively, were sub-cloned by PCR using cDNA clones as templates [10,15]. Primer-adapters were used to incorporate an N-terminal His_6_-tag, and 5'*Nhe*I/3'*Sac*I (132A) or 5'*Nhe*I/3'*Sal*I (159A) restriction sites for sub-cloning into pET21b (Novagen). Final constructs encode recombinant atToc132A and atToc159A including N-terminal hexahistidine tags and are denoted 132A_His _and 159A_His_. The proteins were overexpressed in *E. coli *BL21(DE3) using standard conditions. Cells were lysed in lysis buffer (10 mM Tris-HCl pH 8.0, 50 mM NaCl, 20 mM imidazole) using a French press. Total soluble protein was applied to a Ni^2+^-NTA column (Novagen) at 4°C. The resin was washed with lysis buffer containing 30 mM imidazole, and proteins were eluted with lysis buffer containing 250 mM imidazole. 132A_His _and 159A_His _were further purified using a batch method of ion exchange. Briefly, Ni^2+^-purified protein was diluted 1:1 in ion exchange buffer (20 mM piperazine, pH 4.5, 200 mM NaCl) and incubated with 1.5 mL of a strong-anion exchange resin (Q-Sepharose Fast Flow Ion Exchange Media, GE Health Sciences) for 10 min at room temperature on a rotating mixer. Protein was eluted with a 20 mM piperazine solution at pH 4.5, containing 550 mM NaCl. The purified protein was concentrated and exchanged into CD Buffer (10 mM Tris-HCl, pH 8.0, 50 mM NaCl) using centrifugal diafiltration (Ultracel-10, Millipore). Recombinant 132A_His _and 159A_His _were analyzed by SDS-PAGE (10% resolving gels) stained with Coomassie, or probed on Western blots using antibodies against the A-domain of atToc132 or atToc159 (see below). Protein concentration was determined using the Bradford assay [50], according to the manufacturer's instructions (Bio-Rad).

### Western blots

SDS-PAGE-resolved 132A_His _(~1 μg) and 159A_His _(~10 ng) were transferred to nitrocellulose by first soaking the gel in transfer buffer (12.5 mM Tris, 96 mM glycine, 0.05% (w/v) SDS, and 10% (w/v) methanol). Transfer to nitrocellulose was achieved using a Semi-Dry transfer cell (Bio-Rad Laboratories Inc.) at 15 volts for 90 min. The nitrocellulose was stained with amido black (45% [v/v] methanol, 10% [v/v] acetic acid, 0.1% [w/v] amido black) to confirm successful transfer, and the membrane was blocked by incubating with 5% (w/v) powdered milk in TBS containing 0.1% Tween-20 (TBS-T; 20 mM Tris-HCl pH 7.5, 150 mM NaCl, 0.1% (v/v) Tween-20). The membrane was washed with TBS-T and incubated with primary antibody at room temperature for 2 h. Primary antibodies used were rabbit antibodies raised against the A-domain of atToc159 (α-159A) diluted 1:2000, or the A-domain of atToc132 (α-132A) diluted 1:5000 [10] (gifts from Dr. Danny Schnell, University of Massachusetts). Peroxidase conjugated goat anti-rabbit IgG (Rockland) diluted 1:5000 was used to facilitate chemiluminescent detection. Signal was captured using a Bio-Rad Fluor-S MultiImager in high sensitivity mode, equipped with a Nikkor AF 50 mm lens (Nikon), using an f-stop of 1.4 and an exposure time of 2 to 4 min. The images were analyzed using Quantity One 1-D Analysis software v4.6 (Bio-Rad Laboratories Inc.).

### CD measurements and analysis

Far-UV CD spectra were measured on an Aviv 215 spectropolarimeter (Aviv Biomedical). Measurements were performed using rectangular quartz cells with 0.1 cm pathlength. 132A_His _and 159A_His _were measured at concentrations of 5 μM or 2.5 μM in CD buffer. Samples were equilibrated at the indicated temperature for 10 min prior to measurements, and pH was adjusted immediately prior to measurement for pH-dependent experiments. Spectra of protein samples and the buffer baseline were measured with a 0.5 nm/s scanning speed at 0.5 nm intervals, and were an average of four scans. Averaged buffer baseline spectra were subtracted from averaged protein sample spectra and the resultant corrected spectra were converted to mean residue ellipticity. Spectra were deconvoluted on the Dichroweb website [51] using the K2D method [52].

## Authors' contributions

LGLR participated in planning the study, carried out all the experiments and associated analysis, and drafted the original manuscript. MJ-N participated in the planning and interpretation of the biophysical experiments, and contributed to the preparation of the manuscript. MDS conceived of the study, participated in its design, oversaw the experiments, and helped to draft the manuscript. All authors read and approved of the final manuscript.
